# Basidiomycetes Polysaccharides Regulate Growth and Antioxidant Defense System in Wheat

**DOI:** 10.3390/ijms25136877

**Published:** 2024-06-22

**Authors:** Olga Tsivileva, Andrei Shaternikov, Nina Evseeva

**Affiliations:** Institute of Biochemistry and Physiology of Plants and Microorganisms, Saratov Scientific Centre of the Russian Academy of Sciences, 13 Prospekt Entuziastov, 410049 Saratov, Russia; andrejsh93@gmail.com (A.S.); evseeva_n@ibppm.ru (N.E.)

**Keywords:** wheat plants, *Triticum aestivum* L., higher fungi, mushrooms, xylotrophic basidiomycetes, exogenous substances, natural polymers, fungal exopolysaccharides, growth-stimulating effect, antioxidant defense

## Abstract

Higher-fungi xylotrophic basidiomycetes are known to be the reservoirs of bioactive metabolites. Currently, a great deal of attention has been paid to the exploitation of mycelial fungi products as an innovative alternative in crop protection. No data exist on the mechanisms behind the interaction between xylotrophic mushrooms’ glycopolymeric substances and plants. In this study, the effects of basidiomycete metabolites on the morphophysiological and biochemical variables of wheat plants have been explored. Wheat (*Triticum aestivum* L. cv. Saratovskaya 29) seedlings were treated with extracellular polysaccharides (EPSs) isolated from the submerged cultures of twenty basidiomycete strains assigned to 13 species and 8 genera. The EPS solutions at final concentrations of 15, 40, and 80 mg/L were applied to wheat seedlings followed by their growth for 10 days. In the plant samples, the biomass, length of coleoptile, shoot and root, root number, rate of lipid peroxidation by malondialdehyde concentration, content of hydrogen peroxide, and total phenols were measured. The peroxidase and superoxide dismutase activity were defined. Most of the EPS preparations improved biomass yields, as well as the morphological parameters examined. EPS application enhanced the activities of antioxidant enzymes and decreased oxidative damage to lipids. Judging by its overall effect on the growth indices and redox system of wheat plants, an EPS concentration of 40 mg/L has been shown to be the most beneficial compared to other concentrations. This study proves that novel bioformulations based on mushroom EPSs can be developed and are effective for wheat growth and antioxidative response. Phytostimulating properties found for EPSs give grounds to consider extracellular metabolites produced in the xylotrophic basidiomycete cultures as an active component capable of inducing plant responses to stress.

## 1. Introduction

Reactive oxygen species (ROS), which are by-products of several metabolic processes in plants, are considered the important players that control and regulate the biotic and abiotic stress reactions and development [1]. Various stresses lead to the overproduction of ROS in plants [2]. To remove excessive ROS induced by biotic and abiotic stresses, thus alleviating oxidative damage to plants and the destruction of biological macromolecules, nonenzymatic and enzymatic antioxidant systems are activated. Plants generate a burst of ROS in response to infection by not only virulent but also non-virulent bacteria, fungi, and viruses [3]. Therefore, the exogenic application of cell-free natural products isolated from beneficial microorganisms seems quite reasonable. The fundamental functions and mechanisms of exogenous substances to alleviate stress in plants still need more effort and investments to be fully explored. The action of a number of exogenous substances capable of exerting antioxidative effects could be applied for the efficient scavenging of ROS generated by environmental stressors in wheat [2].

Investigation of ROS metabolism regulation via exogenous substances is of great importance for improving cultured plants’ development and resistance to stress. Substances of a saccharide nature are insufficiently explored exogenous compounds implemented in ROS metabolism regulation that are potentially useful for improving resistance to stress and developing cultivars capable of withstanding various adverse conditions. Sugars are easy to obtain, inexpensive, and can be widely used in agricultural production. Therefore, it is important to analyze the mechanism by which carbohydrates regulate ROS metabolism under stress. For instance, in the studies of exogenous substances that alleviate salt stress in plants, one of the research hot spots has been focused just on saccharides [4]. Antioxidant stimulation as a functional mechanism related to such substances is among recent research focuses.

Carbohydrate-containing monomeric, oligomeric, and polymeric substances can alleviate oxidative damage caused by abiotic stress [5]. Thus, trehalose plays an important role in plant resistance to abiotic stress by enhancing the antioxidant system, activating photosynthesis, and protecting cellular structure [6,7,8]. Polysaccharide chitosan is the only cationic pseudonatural polymer, which is the second most abundant biopolymer in nature [9]. Chitosan is a linear copolymer of glucosamine and acetylglucosamine [10]. Recent studies reported that exogenous chitosan could improve photosynthetic capacity, regulate redox-related enzymatic reactions, and reduce ROS induced by cold stress resistance in plants [11]. Kappa carrageenan (*κ*-carrageenan) is a natural polymer produced commercially, a biocompatible natural sulfated (the degree of sulfation is approximately 6%) polysaccharide extracted from red algae, and an appropriate matrix for polymer nanocomposites [12,13,14]. Carrageenans have been recently assessed as biostimulants and bioelicitors implemented in plant growth and defense responses [15].

The main focus has been beneficial microorganisms offering essential ecological services to both natural and agricultural ecosystems [16]. Glycopolymers are implemented to overcome an oxidative burst during the early stages of plant–bacterial symbiosis. Thus, the levels of production of exopolysaccharides (EPSs) such as succinoglycan and galactoglucan by the bacterium *Sinorhizobium meliloti* were found to correlate positively with survival in hydrogen peroxide [17]. At the initiation of bacterial infection, legume host plants monitor the structure of rhizobial EPSs, which play an active role in signaling to ameliorate plant defense responses, including against host-derived ROS [18]. Comparative evaluation of the influence of distinctly originated EPSs on wheat could broaden the existing knowledge of the mechanisms of EPS-mediated plant–microbial interactions and help to develop effective biofertilizers. Thus, EPSs produced by *Paenibacillus polymyxa*, a polyfunctional plant-growth-promoting rhizobacterium used in agriculture, medicine, and industry, are active metabolites responsible for bacterial contact with wheat roots and are implicated in the induction of plant responses to these interactions [19]. EPSs from *P. polymyxa* 1465 induce plant-protective responses presumably switched on by EPS’ interaction with the protein receptors in the wheat plant cell plasmalemma [20]. Root colonization abilities mediated by beneficial EPSs are considered to be higher because these polymeric materials help rhizobacteria to firmly attach to the root surface even under water-deficit conditions [21]. Under drought stress conditions, EPS-producing bacteria not only improve soil quality by increasing the water-holding capacity but also protect plants [22,23].

There have been many attempts to provide benefits for cultured plants using artificial inoculation with non-wood-decaying arbuscular mycorrhizal fungi (AMF) [24,25]. Arbuscular mycorrhizal symbiosis improves the growth and antioxidative responses of plants under salt stress [26] and regulates microbial dynamics to help plants resist adversities [4]. However, the effect of introducing AMF inoculants to indigenous mycorrhizal communities in the field is difficult to predict. AMF inoculants can persist in the amended areas for a few months to several years, though usually with a decline in abundance over time or even complete extinction [27]. Furthermore, inoculation with AMF can exert both positive and negative impacts on native AMF species, including suppression, stimulation, exclusion, and neutral impacts [16]. The success of interventions with AMF inoculants is dependent on many conditions, such as the inherent inoculum characteristics, the dosage and frequency of inoculation, and the physical and biological factors of soil [28]. Thus, further extensive investigations are required to improve the reproducibility of the positive effects and standardize production processes [29].

Mushrooms are macrofungi that can be classified as basidiomycetes and ascomycetes based on the development patterns in the life cycle [30]. Edible and medicinal xylotrophic mushrooms have long been known to humanity as decomposers in ecosystems, valuable food, and producers of a versatile complex of biologically active substances [31]. Polysaccharides, especially β-glucans, isolated from basidiomycetes have laid the foundation for the invention of new medicines [32,33]. The current research is extremely interested in fungal EPSs due to their highly diverse physiological and biotechnological functions and little or no toxicity. Many, if not all, basidiomycetes accumulate physiologically active EPSs in cultured broth [34,35]. Polysaccharides gained from xylotrophic mushrooms frequently exhibit a broad set of valuable traits, which have driven the interest in research on their functional properties. β-D-glucans have displayed a highly innovative potential, principally related to their desirable biocompatibility accompanied by the readily achievable structural modification mediated by numerous –OH functional groups [36]. Functional peculiarities of β-D-glucans are also closely associated with their physicochemical properties, such as solubility, molecular conformation [37], and branching characteristics [38,39]. Basidiomycetes serve as an enormously rich source of β-D-glucans, of which their distinct phytostimulating effects should, therefore, be screened at the fungal-strain level.

Earlier, we studied the potato response elicited by fungal biopolymeric preparations fortified or not with selenium to form bionanocomposites [40] and found that application of the latter improved the morphometric parameters of potato plants. Despite the fact that xylotrophic basidiomycetes are not a native species for wheat and do not belong to the group of arbuscular mycorrhizal species, the plant-growth-promoting activity of *Laetiporus sulphureus* polysaccharides entering the composition of the fungal cell wall was found very recently for various plant species, including wheat [41]. There is currently little to no information on how EPSs from xylotrophic basidiomycetes affect cultured plants. This study evaluates the effect of EPSs from xylotrophic mushrooms on the growth and antioxidative response of soft spring wheat (*Triticum aestivum* L. cv. Saratovskaya 29). The objective of this work is to perform screening assays to assess twenty strains of xylotrophic basidiomycetes’ extracellular metabolites for their capability to promote growth and regulate the functioning of the pro/antioxidant system in wheat.

## 2. Results

### 2.1. Fungal EPS Features

EPSs were isolated from one-month-old, submerged cultures of xylotrophic mushrooms by following a facile method, therefore the produced basidiomycetes’ extracellular preparations were protein-free glycans [42]. Low-molecular-weight substances, as presumable admixtures in the composition of EPS, could, in principle, be detectable by means of high-performance liquid chromatography (HPLC) [43], but were not revealed. This picture was also supported by the results gained from another chromatographic method implemented, namely, gas chromatography-mass spectrometry (GC-MS). Outcomes regarding the yield of the typical reaction medium after fungal culturing were evidenced [40] by the absence of any appreciable detected substances as admixtures to the EPS chemical composition.

### 2.2. Morphological and Physiological Variables of Wheat Seedlings Exposed to Fungal EPS

The comparison of the morphophysiological variables of the experimental and control plants was indicative of the biological effect of fungal EPSs on seedling growth. The following morphological variables of 10-day-old wheat seedlings were analyzed: coleoptile length, leaf length, root length, and root number (Table 1).

Relative morphometric indices of the 10-day-old wheat seedlings exposed to fungal EPSs are presented in terms of the percentage compared to the control (Table S1). We found that all morphological indices (Table 1 and Table S1) were affected by the EPS treatments. The length of seedling coleoptile was revealed to be 1.51 to 1.02 times higher than that in the control plants, with this positive effect being observed on one-half of the total number of preparations involved in this research (Figure S1).

Coleoptile enlargement was promoted at both 40 and 80 mg/L EPS levels by four fungal preparations, namely *P. ostreatus* 69 (+51% and +19% at 40 and 80 mg/L EPS, respectively), *G. neojaponicum* SIEbidoup (+42% and +35% at 40 and 80 mg/L EPS, respectively), *Laet. sulphureus* (+26% and +13% at 40 and 80 mg/L EPS, respectively), and *Gr. umbellata* (+30% and +2% at 40 and 80 mg/L EPS, respectively). Treatment with the 40 mg/L concentration of EPSs based on *G. colossus* yielded a considerable increase in the coleoptile length of 138% compared to the water-control wheat seedlings, followed by *L. edodes* 198 (115% of control), *G. neojaponicum* SIEbgm (109% of control), *T. cattienensis* (106% of control), and *G. valesiacum* (102% of control). No significant effect of EPSs from *G. neojaponicum* SIEbidoup at the 15 mg/L level on coleoptile length was observed on the 10th day of wheat seedling growth compared to the water control. Obviously, EPS treatment can initiate metabolic changes that lead to improved coleoptile elongation and potentially enhance the deep-sowing tolerance in wheat.

The fungal EPSs produced by ten basidiomycetes strains were capable of enhancing the leaf length parameter in wheat: however, distinct results varying between strains at 15, 40, and 80 mg/L concentrations were obtained (Figure S2). In total, 17 (more than half) modes of experiments concerning this parameter could be regarded as giving positive outcomes, namely *G. neojaponicum* SIEbidoup (+19%, +60%, and +35% at 15, 40, and 80 mg/L EPS, respectively), *P. ostreatus* 69 (+38% and +24% at 40 and 80 mg/L EPS, respectively), *Gr. umbellata* (+37% and +28% at 40 and 80 mg/L EPS, respectively), *L. edodes* F-249 (+37% and +12% at 40 and 80 mg/L EPS, respectively), *F. velutipes* (+34% and +11% at 40 and 80 mg/L EPS, respectively), and *G. colossus* (+18% and +10% at 40 and 80 mg/L EPS, respectively). Seedling growth with 40 mg/L EPSs based on other fungal strains, such as *G. neojaponicum* SIEbgm, *G. valesiacum*, *Laet. sulphureus*, and *G. applanatum* 0154, led to an increase in plant leaf length parameter that exceeded the untreated control by 1.33 times in the case of the biopreparation from *G. neojaponicum* SIEbgm. Leaf length values for plants exposed to the *G. neojaponicum* SIEbidoup-based biopolymer exhibited a substantial increase when the concentration increased from 15 mg/L EPSs to 40 mg/L EPSs (Figure S2).

Remarkable enlargement of the root length in wheat was observed with the *G. neojaponicum* SIEbidoup EPS, with a value of 206% to the control (Figure S3). Root length indices for wheat plants exposed to the mushroom exopolyglycans exceeded the corresponding values of control plants: *G. neojaponicum* SIEbidoup (+106% and +65% at 40 and 80 mg/L EPS, respectively), *G. neojaponicum* SIEbgm (+92% at 40 mg/L EPS), *P. ostreatus* 69 (+61% and +74% at 40 and 80 mg/L EPS, respectively), *F. velutipes* (+57% at 40 mg/L EPS), *G. colossus* (+53% at 40 mg/L EPS), *Gr. umbellata* (+51% and +6% at 40 and 80 mg/L EPS, respectively), and *L. edodes* F-249 (+42% and +37% at 40 and 80 mg/L EPS, respectively). A much weaker, yet still positive, influence on the root length variable in wheat seedlings was found for the 40 mg/L EPS produced by *Armillaria mellea* 0738 (+10%) and *Grifola frondosa* (+2%) compared to the control (Figure S3). Nevertheless, these basidiomycetes are worth special mention as new players among the EPS producers discussed within this research, for which a beneficial effect on the plant’s morphological indices was detected. In general, it suggested increased root length in EPS-treated plants, which contributes to the improvement of the absorption of micro- and macronutrients, accelerating plant growth and stress tolerance formation.

Fungal EPSs exhibited a greater effect on root number when compared to untreated plants, with increments of at least 10% being revealed with exoglycans from *T. cattienensis* (+67%), *G. neojaponicum* SIEbidoup (+45% both at 40 and 80 mg/L EPS), *Gr. umbellata* (+45%), *L. edodes* 198 (+42%), *P. ostreatus* 69 (+42% and +36% at 40 and 80 mg/L EPS, respectively), *G. colossus* (+36%), *Laet. sulphureus* (+36% and +24% at 40 and 80 mg/L EPS, respectively), *P. ostreatus* BK1702 (+18%), *G. lucidum* SIE1303 (+16%), and *L. edodes* F-249 (+11% at 80 mg/L EPS) (Figure S4).

The number of roots did not differ markedly between control and treated plants in the experiments with EPSs derived from *A. mellea* 0738 (+0%), *A. mellea* 1346 (+5%), *F. velutipes* (+4%), and *G. colossus* (+2%). Interestingly, EPSs from *G. neojaponicum* SIEbidoup caused a considerable increase of 145% in wheat root number compared to the experiment, not only at the standard concentration but also at higher (80 mg/L) concentrations in nutrient media, although without losses in its biostimulating effect. These data confirm that several *Ganoderma* species-sourced exoglycans promote wheat growth and development.

**Figure 1:**
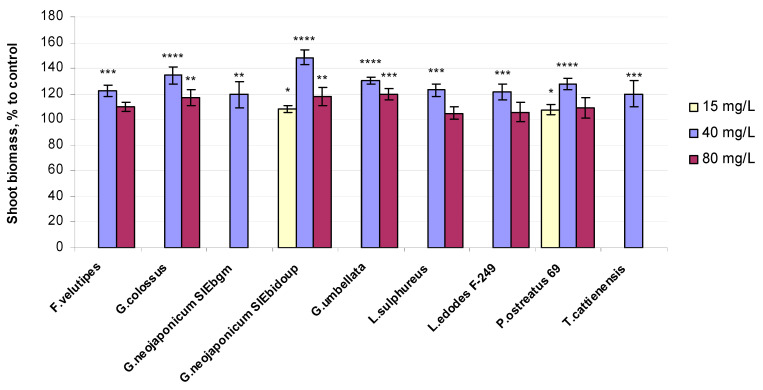
Relative fresh weight values in 10-day-old wheat seedlings exposed to fungal preparations. Values are means ± SD; * *p* ≤ 0,05; ** *p* ≤ 0.005; *** *p* ≤ 0.0005; **** *p* < 0.0001.

Relative shoot biomass values of the 10-day-old wheat seedlings exposed to fungal EPSs are presented in terms of the percentage compared to the control. The experimental choice based on higher values of morphological indices (highlighted in yellow in Table 1 and Table S1) is plotted in the diagram. The results for eighteen EPS samples, thus chosen based on the morphology criterion, are shown in Figure 1.

Shoot fresh weight values for the majority of fungal preparations used were significantly greater in comparison to the unaffected control (Figure 1) and were especially higher in plants exposed to 40 mg/L solutions of EPSs. Preparation from the *Ganoderma* mushroom strain *G. neojaponicum* SIEbidoup showed the maximum increase in shoot fresh weight (+48%) followed by *G. colossus* (+35%), *Gr. umbellata* (+31%), *P. ostreatus* 69 (+28%), *Laet. sulphureus* (+23%), *F. velutipes* (+23%), *L. edodes* F-249 (+22%), *T. cattienensis* (+20%), and *G. neojaponicum* SIEbgm (+19%). Seedlings grown with the lowest EPS concentration (15 mg/L solutions) resulted in the same only slightly improved fresh weight quantities (+8%) (Figure 1). Similar outcomes ranging from 105 to 120 percent of the water-control samples were gained in wheat plants affected by the highest EPS levels: *Gr. umbellata* (+20%), *G. neojaponicum* SIEbidoup (+18%), *G. colossus* (+17%), *F. velutipes* (+10%), *P. ostreatus* 69 (+9%), *L. edodes* F-249 (+6%), and *Laet. sulphureus* (+5%).

Wheat seedlings grown in the nutrient media with EPSs produced by the oyster mushroom strain *P. ostreatus* 69 had the same value of shoot fresh biomass at both 15 mg/L and 80 mg/L levels of fungal preparation, while the maximum value with this strain was shown under the influence of 40 mg/L EPSs (+28%), although this was considerably lower than for *G. neojaponicum* SIEbidoup EPSs (+48%) (Figure 1).

### 2.3. Biochemical Variables of Wheat Seedlings Exposed to Fungal EPS

#### 2.3.1. Lipid Peroxidation Status

For the evaluation of biochemical characteristics of wheat plants, samples of fresh leaves were taken from each treatment for the estimation of enzymatic and non-enzymatic antioxidants, hydrogen peroxide (H_2_O_2_), and malonaldehyde (MDA) contents. To elucidate the antioxidative effect of the fungal exoglycans in question, we examine if EPSs cause the accumulation of one of the main final products of lipid peroxidation, MDA, in the shoots. A higher MDA level is consistent with lipid peroxidation intensity and indicates more severe membrane damage.

**Figure 2:**
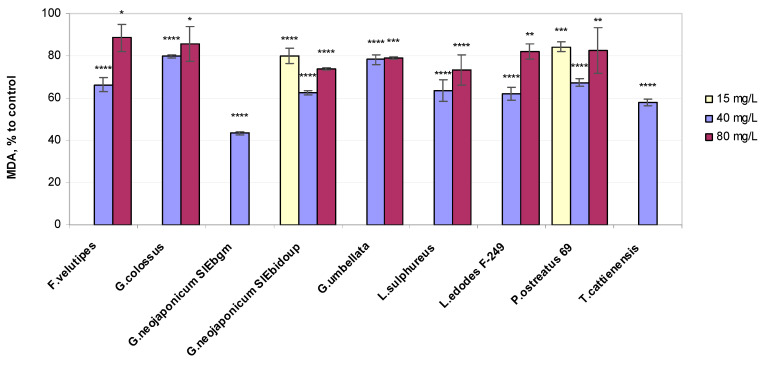
Relative MDA values in 10-day-old wheat seedlings exposed to fungal preparations. Values are means ± SD; * *p* ≤ 0,05; ** *p* ≤ 0.005; *** *p* ≤ 0.0005; **** *p* < 0.0001.

Fungal EPS treatments in the wheat plants led to a reduction in MDA content as compared to the water-control seedlings (Figure 2).

A significant reduction in the MDA concentration, which yielded relative quantities of 43% to 80% of the control, was detected in wheat seedlings exposed to 40 mg/L EPS preparations: *G. neojaponicum* SIEbgm (43%), *T. cattienensis* (58%), *G. neojaponicum* SIEbidoup (62%), *L. edodes* F-249 (62%), *Laet. sulphureus* (64%), *F. velutipes* (66%), *P. ostreatus* 69 (67%), *Gr. umbellata* (78%), and *G. colossus* (80%). However, the seedlings exposed to 80 mg/L EPSs biosynthesized by *F. velutipes* (−11%), *G. colossus* (−14%), *L. edodes* F-249 (−18%), and *P. ostreatus* 69 (−18%) were characterized by more elevated levels of lipid peroxidation yet were lower than the control (Figure 2). These data indicated that at the cellular level, plants treated with all the mushroom exoglycans in question were better protected against oxidative damage. This may be related to their direct antioxidant effect. In response to stresses, the production of ROS sharply increases in plants, which results in increased lipid peroxidation of cell membrane structures, disruption of their integrity, and, as a consequence, a change in the barrier properties of cell membranes [44]. The application of fungal EPSs significantly reduced lipid peroxidation in wheat plants.

We carried out Pearson correlation analysis, the results of which indicated that growth parameters in a number of cases were significantly associated with metabolic activity (Table 2). For each of the three groups within the whole preparation set arranged based on a concentration rate, strong positive or negative relationships were shown to be significant (*p* < 0.05) in 11 cases. From the whole set of fungal preparations, the Pearson correlation coefficient (R) was negative in 50% of cases for root number comparisons with the MDA level, with R varying from −0.9933 to −0.7244 at 0.0211 < *p* < 0.0368.

#### 2.3.2. Total Phenols Content

**Figure 3:**
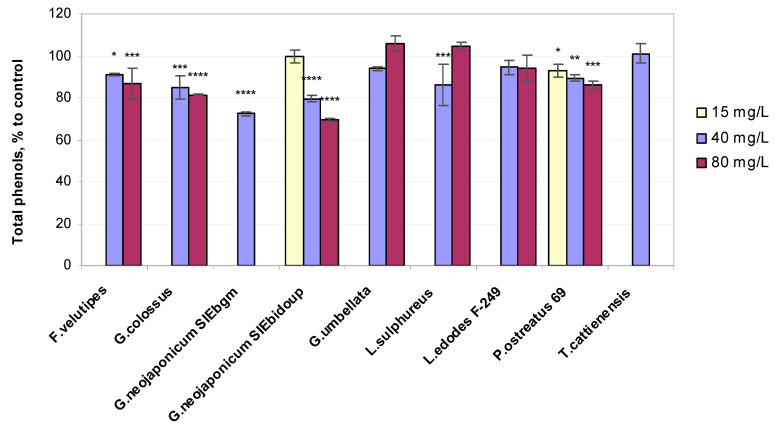
Relative total phenol content values in 10-day-old wheat seedlings exposed to fungal preparations. Values are means ± SD; * *p* ≤ 0,05; ** *p* ≤ 0.005; *** *p* ≤ 0.0005; **** *p* < 0.0001.

The effect of fungal EPSs on the phenolic content regarding nonenzymatic compounds as components of the pro/antioxidative system in wheat was determined (Figure 3).

The total phenolic content values in wheat seedlings exposed to fungal EPSs increased in several modes of the experiment and were only slightly higher than the control: *Gr. umbellata* (+6% at 80 mg/L EPS), *Laet. sulphureus* (+5% at 80 mg/L EPS), and *T. cattienensis* (+1% at 40 mg/L EPS). The other total phenolic quantities in plants do not exceed the water controls. Treatment with the EPS derived from *G. neojaponicum* SIEbidoup displayed the maximum decrease in the total phenol level of shoots compared to the untreated control (−31% at 80 mg/L EPS). The fungal EPS preparation obtained from *P. ostreatus* 69 appeared to cause a response in wheat seedlings’ phenolics level even at the lowest tested concentration of 15 mg/L EPSs (−7%) (Figure 3). In general, there was a noticeable concentration dependence for the results of phenolic content measured in wheat seedlings under the influence of different fungal EPSs, with the exclusion of the EPS derived from *L. edodes* F-249. In the latter mode of the experiment, the plant phenols level remained at virtually the same level of 95% compared to the control. Correlation analysis indicated (Table 2) that the correlation coefficient was negative in 89% of cases for root length comparisons with the phenol level, with R varying from −0.8190 to −0.7406 at 0.0064 < *p*<0.0071. Leaf length negatively (R = −0.6455) and significantly (*p* = 0.0419) correlated with total phenols for 39% of fungal preparations.

#### 2.3.3. Hydrogen Peroxide Level

**Figure 4:**
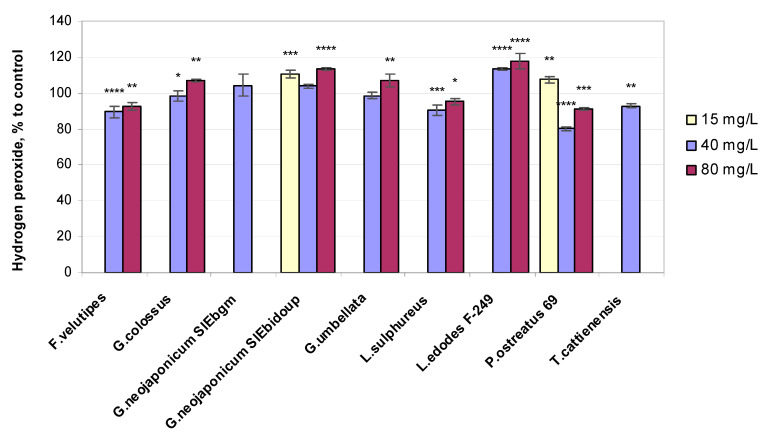
Relative hydrogen peroxide level values in 10-day-old wheat seedlings exposed to fungal preparations. Values are means ± SD; * *p* ≤ 0,05; ** *p* ≤ 0.005; *** *p* ≤ 0.0005; **** *p* < 0.0001.

Hydrogen peroxide, a non-radical ROS, has been characterized as the leading plant biomarker of oxidative damage [45]. Treatment with fungal EPSs maintained the hydrogen peroxide concentration in the leaves near the baseline in the majority of experimental modes (Figure 4).

H_2_O_2_ concentration values in shoots were marginally higher in comparison to the unaffected control for half of all the fungal EPS preparations used (Figure 4): *L. edodes* F-249 (+14% and +18% at 40 and 80 mg/L EPS, respectively), *G. neojaponicum* SIEbidoup (+11%, +4%, and +14% at 15, 40, and 80 mg/L EPS, respectively), *Gr. umbellata* and *G. colossus* (+7% at 80 mg/L), *P. ostreatus* 69 (+7% at 15 mg/L), and *G. neojaponicum* SIEbgm (+4% at 40 mg/L). Hydrogen peroxide content increased by 14–18% in the assay with *L. edodes* F-249-derived EPSs (Figure 4). The minimum hydrogen peroxide concentration was recorded with *P. ostreatus* 69 (0.80 of the control) followed by *F. velutipes* (0.89), *Laet. sulphureus* (0.90), *T. cattienensis* (0.93), *G. colossus* (0.98), and *Gr. umbellata* (0.99). In this respect, there was no significant difference between the control and plants treated with the *G. colossus-* and *Gr. umbellata*-based preparations. All listed values increased only slightly to negligibly by 1.03–1.14 times when increasing from the middle to the highest examined EPS concentrations.

Hydrogen peroxide levels exhibited high positive correlations with two morphological indices (Table 2), leaf length (R = 0.9881), and root length (R = 0.9980). This correlation was considered to be statistically significant (*p* values equal to 0.0492 and 0.0202, respectively) in only 11% of fungal preparations.

#### 2.3.4. Enzyme-Related Bioassays

Several common, well-studied enzymes with suspected or well-documented roles in the plant antistress defense mechanism include peroxidase (POx) and superoxidedismutase (SOD). Many authors note that environmental stress and antioxidant enzyme activity have significant interactions, and inoculation with EPS-producing bacteria alleviates the adverse effect of stressful conditions on the antioxidant enzyme activity [46,47]. In attempting to gain supportive evidence for fungal EPS, the plant–biomarker assays could be addressed, including measuring POx and SOD activities.

**Figure 5:**
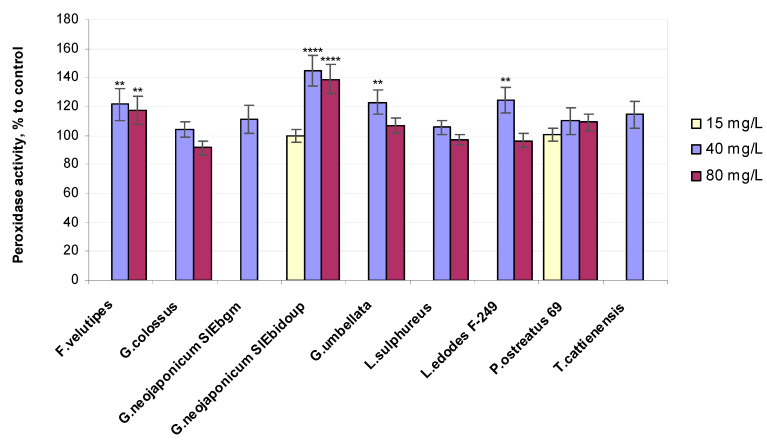
Relative peroxidase activity values in 10-day-old wheat seedlings exposed to fungal preparations. Values are means ± SD; ** *p* ≤ 0.005; **** *p* < 0.0001.

POx activity in plants was practically unaffected by the lowest 15 mg/L concentration of fungal EPSs implemented (Figure 5).

**Figure 6:**
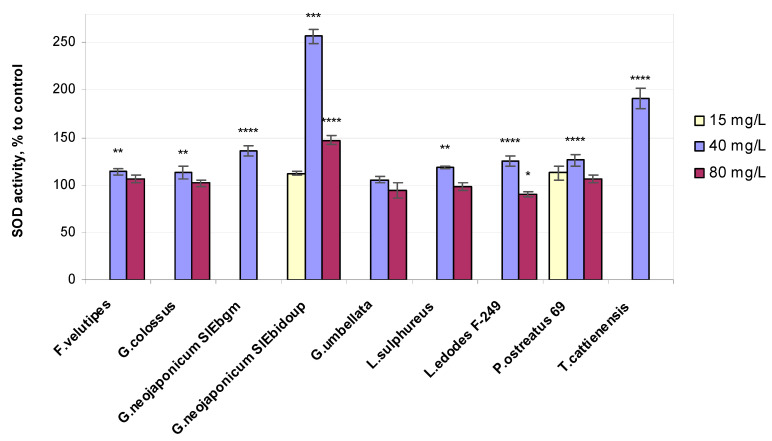
Relative superoxide dismutase activity values in 10-day-old wheat seedlings exposed to fungal preparations. Values are means ± SD; * *p* ≤ 0,05; ** *p* ≤ 0.005; *** *p* ≤ 0.0005; **** *p* < 0.0001.

In contrast, the POx variable was much greater in plants treated with the higher concentrations of mushroom EPS: *G. colossus* (+4% POx), *Laet. sulphureus* (+5% POx), *P. ostreatus* 69 (+10% POx), *G. neojaponicum* SIEbgm (+11% POx), *T. cattienensis* (+14% POx), *F. velutipes* (+21% POx), *Gr. umbellata* (+23% POx), *L. edodes* F-249 (+25% POx), and *G. neojaponicum* SIEbidoup (+44% POx). The relative values of superoxide dismutase activity in wheat plants exposed to fungal EPSs are depicted in Figure 6.

In terms of the higher activity of POx and SOD induced in wheat plants by the applied fungal EPSs, the highest ranking of the entire experiment should be assigned to EPSs from *G. neojaponicum* SIEbidoup (+157% SOD, +44% POx), followed by *T. cattienensis* (+92% SOD, +14% POx), *G. neojaponicum* SIEbgm (+36% SOD, +11% POx), *P. ostreatus* 69 (+26% SOD, +10% POx), *L. edodes* F-249 (+25% SOD, +25% POx), *Laet. sulphureus* (+18% SOD, +5% POx), *F. velutipes* (+14% SOD, +21% Pox), *G. colossus* (+14% SOD, +4% Pox), and *Gr. umbellata* (+5% SOD, +23% POx). Within the entire experiment, regarding the seedling growth supplemented with fungal EPSs, SOD-specific activity varied from 257% down to 105% of the control for *G. neojaponicum* SIEbidoup and *Gr. umbellata* EPS, respectively. Pox-specific activity experienced lower-amplitude changes, within the range of 144% down to 104% for *G. neojaponicum* SIEbidoup and *G. colossus* EPS, respectively. A positive significant (*p* = 0.0153) correlation (R = 0.7543) was observed between POx activity and root length values in 39% of cases with such comparisons (Table 2). A strong positive relationship was found between SOD activity and morphometric parameters: coleoptile length (R = 0.9962), root length (R = 0.7135), and root number (R = 0.9897). The correlation was significant, with *p*-values equal to 0.0277, 0.0234, and 0.0457, respectively, and was observed in 50% of cases with these comparisons.

## 3. Discussion

### 3.1. Morphology-Related Response in Wheat Plants Exposed to Fungal EPS

Wheat (*Triticum aestivum* L.) is among the most important staple food crops globally [48,49] and is widely cultivated in many countries. The sustainability of wheat production is of great interest to food security. The use of exogenous substances to regulate the generation and scavenging of ROS in plants has been widely studied to lay the foundations for alleviating oxidative damage caused by biotic and abiotic stresses in cultivated plants [50]. Furthermore, the exogenously applied physiologically valuable chemical compounds effective at low concentrations could improve the plant water status and mitigate the negative effects of chloride salinity on wheat plant growth [51]. We evaluated the effect of mushroom EPSs on the morphophysiological and biochemical characteristics of wheat seedlings, with the example of twenty EPSs isolated from xylotrophic basidiomycetes-submerged cultures and introduced into a nutrient medium of wheat (*T. aestivum* L.).

Growth could be improved through the plant–EPS interaction with the allowance for colonizing roots more efficiently under environmental stress conditions. When inoculated into the rhizospheres of wheat (*Triticum aestivum*) and maize (*Zea mays*) seedlings, EPS-producing rhizobacteria succeeded in delaying the onset of plant drought symptoms [52]. Responding to drought stress is closely linked to alterations in the root system architecture. The wheat–EPS interaction proceeds with similar consequences. In particular, in wheat, EPS-producing rhizobacteria significantly increased root branching, root length, and root number compared to the non-inoculated control [53].

The coleoptile is an important trait for increasing drought tolerance in wheat, which is essential for plant establishment [54]. This is why special attention has been paid to coleoptile traits and the consequences of greater coleoptile length in wheat breeding programs. Drought is a prevalent natural factor limiting crop production in arid regions across the world. To overcome this limitation, seeds are sown much deeper to boost germination via soil moisture produced by underground water [55]. Wheat coleoptiles protect the plumule and the first leaf so they can move from the embryo to the soil surface. Cultivars with longer coleoptiles are sown deeper and are more successful under drought-stress conditions [56]. In our research, the coleoptile length parameter in wheat seedlings was mainly enlarged, compared to the control, with a 40 mg/L EPS concentration and with basidiomycetes’ exoglycans such as *P. ostreatus* 69 (+51%), *G. neojaponicum* SIEbidoup (+42%), and *G. colossus* (+38%). It can be seen from Figure S1 that only a few of our experimental outcomes display an explicitly positive impact of the 80 mg/L-concentration of mushroom EPSs on wheat coleoptile length, but the EPSs from *G. neojaponicum* SIEbidoup (+35%), *P. ostreatus* 69 (+19%), and *Laet. sulphureus* (+13%) do possess this trait.

Morphophysiological foliar traits of wheat seedlings linked to photosynthetic and growth performance can reflect the adaptability of the plant to the environment [57]. The development of optimal leaf morphology is important for daily carbon gain, thus being closely associated with photosynthetic capacity, the photosynthetic rate, and biomass yield [35,58]. This knowledge could ultimately help to identify opportunities to improve wheat yield in stressful environments [59]. All treatments carried out with the *G. neojaponicum* SIEbidoup-derived exoglycans resulted in increased leaf length within the entire concentration range of EPSs applied to wheat plants compared to the non-treated control. At the optimal EPS concentration, by the end of the observation period, the wheat leaves were approximately 1.60 times longer than the control seedlings. Moreover, the leaf length values (Figure S2) considerably increased as a result of exposure to the basidiomycetes’ EPSs based on *P. ostreatus* 69 (138% to control), *Gr. umbellata* (137% to control), *L. edodes* F-249 (137% to control), *F. velutipes* (134% to control), *G. neojaponicum* SIEbgm (133% to control), *G. colossus* (118% to control), and *G. valesiacum* (118% to control). Despite not being a severely effective stimulating agent with respect to the wheat plants studied, the EPSs obtained from the mushroom *G. valesiacum* were superior at improving the leaf length variable in seedlings compared to the control.

Prospective mechanisms underlying the fungal EPSs action for advanced plant growth may be linked to changes in the root architecture, specifically elongation, because longer roots that penetrate into deeper soil layers tend to provide plants with more water and, thus, better growth and development under normal and stressful conditions. The treatment of seedlings with fungal EPSs also contributed to greater total root length compared to the control. Nine fungal strains, from which thirteen preparations with different EPS concentrations were obtained, all exhibited a positive impact on the wheat root length parameter, which varied from 102% to 206% compared to water-control seedlings (Figure S3). An outstanding increase in this morphological index equal to 106% was observed for the *G. neojaponicum* SIEbidoup EPS. The prevailing tendency of root length values to be higher with exposure of the plant to 40 mg/L-levels of the given fungal EPSs in comparison to 80 mg/L-levels was preserved with EPS producers such as *G. neojaponicum* SIEbidoup, *Gr. umbellata*, and *L. edodes* F-249. These polysaccharides’ unambiguously beneficial effects essentially decreased at the 80 mg/L EPS concentration. In contrast, the impact of the *P. ostreatus* 69-based glycopolymer on the root length index (161% to control) was enhanced at the 80 mg/L level and exceeded the control by 1.74 times (Figure S3).

The occurrence of greater linear root length and more root tips has been previously correlated with better water stress tolerance and overall improvements in maintaining plant productivity under drought [60]. Root system length contributes to more effective soil exploration, whereas the increased number of roots, resulting in more root tips, is obviously of importance for root water uptake capacity [61,62]. The researchers noted that the improved root characteristics of bacterial EPS-exposed wheat seedlings may enable rapid resource acquisition through the expansion of the root system [63]. The positive influence of the majority of fungal EPSs on wheat plant growth when comparing the seedlings’ morphological indices with the untreated control was explicitly pronounced as an increase in the number of roots. Application of the *T. cattienensis-*, *G. neojaponicum* SIEbidoup-, and *Gr. umbellata*-derived EPSs at the 40 mg/L level or EPSs produced in *G. neojaponicum* SIEbidoup at an 80 mg/L-concentration showed the greatest increase in root number, falling in the range of 45–67% compared to the non-treated control (Figure S4). Significantly increased root length and number of roots when compared to the control indicates a more versatile capability of withstanding water-deficit conditions and other stressing factors. Water-stress tolerance results, in large part, from useful changes in the root system architecture [52]. This is why the quantitative assessment of wheat performance across the mushroom EPS-supplementation experiments generally suggests that these EPS-mediated changes in root system variables may have led to much more reliable avoidance of drought stress symptoms.

### 3.2. Biomass-Related Response in Wheat Plants Exposed to Fungal EPS

Key aspects in estimating the effect of different phytostimulants are commonly recognized to include shoot mass [64]. We evaluated the quantities of plant material biomass (Figure 1), which appeared to be distinct for different mushroom producers of EPSs used for seedlings treatment. The overall tendency for the shoot fresh weight of plants exposed to all fungal EPSs to be higher in comparison to the untreated control was preserved at all EPS levels examined. This may be owing to the fact that EPSs influence the growth of plants through various mechanisms, and many studies have reported the effect of EPS-producing bacteria inoculation on plant health in various crops [65]. For instance, seed bacterization with EPS-producing bacterial strains in combination with their respective EPSs improved plant biomass, shoot and root length, and leaf area in maize [66]. In general, EPSs are capable of water holding, due to which bacterial EPSs participate in the maintenance of not only the moisture content of soil but also the flow of water across the plant roots [67]. Greater amounts of extracellular polymeric substances were observed with soil bacteria subjected to desiccation, which could provide much slower water loss from soil and maintain water phase continuity in the soil even under drought conditions, thereby enabling the diffusion of nutrients [67]. EPSs of the oyster mushroom strain *P. ostreatus* 69 yielded similar values of the fresh weight of shoots in both the lowest and highest EPS concentrations, which show a moderate response effect for these exoglycans regarding the physiological indices of wheat plants (Figure 1). One could also identify the positive correlations between *Ganoderma* EPS loading and shoot fresh weight, essentially regardless of the EPS concentration used for treatment, further demonstrating the potential utility of extracellular glycans biosynthesized by *G. neojaponicum* and *G. colossus* in future applications. Inoculation by beneficial microorganisms enhances biomass production along with enzymatic activity (SOD and POx) [68]. Enhanced yield and POx activity were observed in soybeans (*Glycine max*) inoculated with AMF and *Bradyrhizobium japonicum* to mitigate drought stress [69].

### 3.3. Non-Enzymatic Antioxidant-Related Response in Wheat Plants Exposed to Fungal EPS

The complex supplementation of the culture medium with elicitors, precursors, and other functional substances including polysaccharides may significantly enhance growth variables but can also improve pro/antioxidant system functioning in plants [70]. The promotion of antioxidant enzyme activity constitutes one of the modes of action associated with the exogenous substances used in plants to alleviate stress [71]. The increased production of antioxidants has been found to be among the mechanisms through which plant responses to primary abiotic factors pose a threat to the growth and development of crops.

To better understand the mechanisms of fungal EPS functioning in wheat, we also assessed the effects on biochemical levels by measuring ROS production and lipid peroxidation (via MDA). H_2_O_2_ combines the properties of a stress metabolite and a signaling molecule and is considered a secondary messenger in the signal cascade that turns on the induction of the synthesis of antioxidant enzymes [72]. Our results revealed that MDA concentrations were significantly decreased in plants treated with the majority of EPSs obtained from the xylotrophic mushroom cultures we examined. Based on the results depicted in Figure 2, it can be concluded that in all fungal EPS-exposed wheat plants, the content of MDA decreased by values ranging from 11% (with *F. velutipes* EPSs at 80 mg/L) to 57% (with *G. neojaponicum* SIEbgm EPSs at 40 mg/L) compared to the controls. This seems generally consistent with the current views on lipid peroxidation in wheat. MDA content is recognized as an indicator characterizing the state of the wheat plant cell membrane [73]. EPS-producing bacteria promote plant growth in both abiotically stressed and unstressed environments by means of reducing the leaf content of MDA, thereby neutralizing oxidative damage to plant cells [74,75]. Data on the growth-stimulating activity of beneficial bacteria coincide with the effect we observed concerning decreased lipid peroxidation in the fungal EPS-treated wheat. This has been confirmed by contemporary research on the MDA changes model in plants in response to stressful environmental stimuli. Thus, in drought-stressed wheat, the MDA level increased by 246% in shoots compared to the control plants, whereas the inoculation with the drought-tolerant bacterium *Bacillus megaterium* showed a drastically decreased level of MDA contents in shoots of 57as compared to non-treated water-stressed plants [76]. In our research, the wheat seedlings’ growth process, which was supplemented by EPSs biosynthesized by *F. velutipes*, *Laet. sulphureus*, *P. ostreatus* 69, and *T. cattienensis* at optimal and higher EPS concentrations, was accompanied by a profound decrease in the content of two major non-enzymatic indicators of oxidative stress in plants: MDA (Figure 2), the final product of lipid peroxidation, and hydrogen peroxide (Figure 4), an important ROS. Furthermore, one should regard the near-10% variation in H_2_O_2_ content as a factor that makes a negligible difference, taking note of the several-fold increase in hydrogen peroxide content during an oxidative burst in plants. Two distinct physiological responses generated by H_2_O_2_ levels are already recognized: high concentrations of H_2_O_2_ ultimately trigger cell death, whereas low levels of H_2_O_2_ function as a signal to initiate the plant’s protective responses [77]. The latter seems to be the case for the wheat seedlings exposed to fungal EPSs.

During adverse conditions, plants induce the biosynthesis of phenolic compounds to provide tolerance [78]. Studies have indicated that the phenylpropanoid pathway is the most involved pathway in response to biotic and abiotic stress [79]. In this pathway, plant enzymes, such as phenylalanine ammonia lyase, are activated under stress conditions to synthesize phenolic compounds [80]. Therefore, phenolics are metabolites that play a pivotal role in the defense against biotic and abiotic stresses by mitigating oxidative stress. Very recent findings expanded our knowledge of the response mechanisms to abiotic stress, particularly regarding the regulation of key phenolic biosynthetic genes in rapeseed [81], wherein a higher polyphenolic compound content in plants under drought stress conditions was revealed. It was found that SOD, POx, and polyphenols, along with alkaloids, in plants respond to salt stress to scavenge ROS [82].

Phenolic compounds’ polymerization is triggered by ROS under stress conditions when the formation of the rigid cell wall is accelerated [83]. EPS-producing bacteria, e.g., *Pseudomonas aeruginosa*, a potential stress-tolerant biocontrol agent [84], and *Bacillus megaterium,* protected maize from salinity-caused injury, improved plant growth, and enhanced phenols and antioxidant enzymes, also through the lignification of cell walls [85]. Salt stress led to the intensification of lignin deposition in bean roots [74]. In accordance with the literature data, increased lignin deposition during stress has been demonstrated for different plant species.

The EPSs biosynthesized by *Gr. umbellata*, *L. sulphureus,* and, to a lesser extent, *T. cattienensis* somewhat enhanced phenol content in plants under normal conditions. The efficiency of EPSs from *Gr. umbellata* and *L. sulphureus* in the intensification of phenolics biosynthesis in wheat seedlings differs from other basidiomycetes’ EPSs in our research, indicating the specificity of the fungal polysaccharidic preparations’ action. Those differences are most likely related to the fact that different EPSs contain a different spectrum (and/or number) of specific bioactive glycan moieties involved in the formation of interactions with plants under normal environmental conditions [86]. The substantially elevated level of phenolic compounds during oxidative stress potentially protects plants from oxidative damage [87]. We were unable to detect any significant increment (exceeding the 6% mentioned above) in the total phenolics content in plants as a consequence of EPS treatment, which may be indicative of these fungal polyglycans exhibiting very moderate, if any, adverse impact on wheat seedlings.

A potential protective function of EPSs against hydrogen peroxide during the early stages of host-beneficial bacteria interactions is well known [88]. Many data support that hydrogen peroxide acts as a signaling molecule in the activation of antioxidant enzyme regulation. Greater H_2_O_2_ accumulation could be consequently induced in seedlings characterized by significantly lower POx but higher SOD activities. Cetinel et al. observed similar consequences in an H_2_O_2_-treated group of wheat plants compared to controls under the same conditions [55].

Under the influence of fungal EPSs, there was an increase in the content of hydrogen peroxide in the seedlings, accompanied by an increase in the activity of antioxidant enzymes: superoxide dismutase and peroxidase. Plants’ exposure to the EPS derived from *L. edodes* F-249 resulted in the highest concentrations of H_2_O_2_ detected in shoots within the whole experiment (+14% and +18% at 40 and 80 mg/L EPS, respectively, vs. control). This tendency of the *L. edodes* F-249 strain-produced EPS to follow a direct concentration dependence in enhancing hydrogen peroxide levels in wheat seedlings (Figure 4) could be related to the capability of these EPSs to support the phenolics content in the same plants being essentially unchanged (Figure 3).

The reduction in MDA and H_2_O_2_ concentrations could be attributed to the enhanced capacity of the antioxidant system to scavenge ROS, which prevented the cells from oxidative damage [48]. These findings are in agreement with the results of Wang et al. [89], who pointed out that H_2_O_2_ acts as a signaling molecule capable of activating antioxidant defense mechanisms in plants, decreasing the level of lipid peroxidation, and thus, reducing MDA concentrations.

The present study on wheat plants has demonstrated that the seedlings treated with the *P. ostreatus* 69 strain EPS exhibited the greatest loss in the H_2_O_2_ concentration of the whole experiment, with a value of 20% compared to the control at the optimal EPS level (Figure 4), and therefore restricted cellular oxidative damage. This finding is in agreement with the rather high sensitivity of wheat seedlings’ antioxidant content to this mushroom EPS, with the latter inducing a response in plants’ total phenol concentrations even at the lowest tested level of 15 mg/L EPSs (Figure 3). Hydrogen peroxide, at high concentrations, may damage lipid membranes [17]. At the same time, the modulation of antioxidant defense processes with hydrogen peroxide may improve the growth of winter wheat (*Triticum aestivum* L.) plants [48] under varied abiotic stresses [55]. Differences in wheat varieties and the hormonal traits exhibited by H_2_O_2_ frequently lead to opposing results related to the development of hydrogen peroxide-affected plants. The signaling function of the H_2_O_2_ molecule is also widely reported [1,90], which is capable of regulating stress tolerance mechanisms in plants [91].

### 3.4. Enzymatic Antioxidant-Related Response in Wheat Plants Exposed to Fungal EPS

A wide range of environmental factors selectively influence plant biomass growth, the yield of secondary metabolites, and the plant enzyme-related redox status. For instance, under water-deficit conditions, the beneficial microorganism inoculations reinforce the antioxidant defense machinery leading to reduced hydrogen peroxide and malondialdehyde content, while boosting the activity of enzymes such as superoxide dismutase and peroxidase [92,93].

SOD is considered the first key line of defense to remove active oxygen in plants. This principal ROS-scavenging enzyme can scavenge superoxide radical anions and generate H_2_O_2_, whereas POx assists in neutralizing the H_2_O_2_ produced during SOD interaction with ROS [45]. An imbalance between the production of ROS and their detoxification at the cellular level leads to oxidative damage under exposure to a stressful environment [94]. Hydrogen peroxide is involved not only in growth processes in plants but also in damage mechanisms [72]. Fungal EPSs caused strongly positive SOD activity changes in 10-day-old plants, which supported the hydrogen peroxide content in leaves at a level slightly exceeding that of the control: *G. neojaponicum* SIEbidoup (+4% H_2_O_2_, +157% SOD, +44% POx), *G. neojaponicum* SIEbgm (+4% H_2_O_2_, +36% SOD, +11% POx), *P. ostreatus* 69 (+7% H_2_O_2_, +26% SOD, +10% POx), and *L. edodes* F-249 (+14% H_2_O_2_, +25% SOD, +25% POx). This could be explained by the fact that as the first line of defense, SOD already played its role in superoxide radical scavenging, which in turn resulted in hydrogen peroxide production.

Fungal EPSs causing moderate SOD activity changes in 10-day-old plants and exceeding the corresponding control values decreased the hydrogen peroxide content in leaves: *Laet. sulphureus* (−10% H_2_O_2_, +18% SOD), *F. velutipes* (−11% H_2_O_2_, +14% SOD), *G. colossus* (−2% H_2_O_2_, +14% SOD), and *Gr. umbellata* (−1% H_2_O_2_, +5% SOD). Treatment with the *T. cattienensis* EPS also resulted in a decrease in hydrogen peroxide (−7% H_2_O_2_) in leaves; however, the enzymatic activity was much greater (+92% SOD). The results of the same experiment show that POx activity levels in plants were not significantly affected by exposure to the above-listed EPS preparations when compared to control groups: *G. colossus* (+4% POx), *Laet. sulphureus* (+5% POx), *T. cattienensis* (+14% POx), *F. velutipes* (+21% POx), and *Gr. umbellata* (+23% POx).

Peroxidases are multifunctional redox enzymes that regulate biochemical processes such as the cross-linking of cell wall components, the production of secondary metabolites, and key reactions in the hypersensitive response [95]. POx reduces hydrogen peroxide to water on account of phenolic oxidation as a substrate-donor of electrons and provides a normal course of oxidation processes as different negative influences on plants. Most researchers associate POx activation with the improved function of the pro/antioxidant system in plants. POx is a good stress marker, but its functions are not restricted by the elimination of hydrogen peroxide. Under certain conditions (in particular, polyphenol oxidation), some apoplastic peroxidases exhibit their oxidase activity via the production of the superoxide radical anion and H_2_O_2_ at physiological pH values [96]. When a plant is impacted by a stressful environment, POx can play the role of either an anti- or prooxidant. Challenges also appear concerning the possible mechanisms and factors providing the stimulation of the ROS-producing activity of POx.

Many studies have suggested a dual role of ROS in plant biology as both toxic byproducts of aerobic metabolism and key regulators of growth, development, and defense pathways [1,90]. ROS act as important signal transduction molecules during the stress response, plant growth, and development; however, excessive ROS accumulate in cells during different stress conditions in the form of toxic metabolites [55]. The superoxide radical anion-producing ability of POx was supposed to rely on phenolic compounds as both peroxidase substrates and possible factors enhancing free radical production [96].

It would be of interest to verify plant phenol implementation in the SOD–POx relationship in wheat shoots in terms of the changes in the hydrogen peroxide concentration. In our research, minimal SOD-specific activity in plants was displayed with *Gr. umbellata*-derived EPSs, with a value of only +5%, accompanied by a rather high increment in the H_2_O_2_ level (+7%) and POx activity (+23%). Essentially the same level of leaf POx (Figure 5) was observed when applying *L. edodes* F-249-derived EPSs (+25%), accompanied by the highest H_2_O_2_ content of the whole experiment (114–118% of control) and elevated SOD activity (+25%), in contrast to the assays with *Gr. umbellata* EPSs (Figure 6). The total phenol values in wheat seedlings exposed to *L. edodes* F-249 strain EPSs remained virtually unchanged (95% of control) when increasing from 40 to 80 mg/L EPS concentrations, whereas *Gr. umbellata* EPSs exhibited the capability of enhancing the total phenol content in plants, which was the greatest we observed with fungal EPSs (Figure 3).

Phenolic compounds, being electron donors and reductants for POx by nature, are one-electron aromatic substrates of plant peroxidases. There is a chance that phenols can serve as the main activators in superoxide formation by POx on the cell surface [97]. The peroxidase system implicating POx + H_2_O_2_ + phenolcarboxylic acids possesses a high radical-producing ability and is known to form ROS. The phenolic radicals oxidized in POx-dependent reactions are supposed to undergo auto-oxidation to form the superoxide radical anion and H_2_O_2_. Therefore, some phenolic compounds may be the source of ROS themselves [98], e.g., the formation of the superoxide radical anion may be the result of the auto-oxidation of a number of phenolic compounds found in plants [99]. As for the exogenous application of natural phenols to plants, according to the data obtained by Aksenova et al. [97], the effect of phenolic precursors (L-phenylalanine, trans-cinnamic acid, and naringenin) on *Camellia sinensis* callus cultures did not lead to significant increases in SOD and POx activity in most cases.

As a result, EPS loading contributed to the regulation of wheat’s pro/antioxidant system, leading to the better development of plants. This is evidenced by the higher values of the morphological and physiological variables of the treated plants compared to the control indices (Table 1 and Table S1, Figure 1, Figure 2, Figure 3, Figure 4, Figure 5, Figure 6 and Figures S1–S4). The obtained data also indicate the fungal strain-dependent efficiency of EPSs since these preparations differently improved growth and biochemical response reactions of the same wheat plants. This phenomenon requires further careful study for a more comprehensive use of fungal EPSs’ potentiality in environmentally oriented technologies for growing wheat, especially under adverse stresses.

## 4. Materials and Methods

### 4.1. Fungal Material and Preparations

Twenty basidiomycetes strains assigned to 13 species from 8 genera were used to obtain the fungal preparations. The cultures included *Armillaria mellea* (Vahl) P. Kumm., strains 0738 and 1346; *Flammulina velutipes* (Curtis) Singer, strain 0535; *Ganoderma lucidum* (Curtis) P. Karst., strain 1315; *Ganoderma applanatum* (Persoon) Patouillard, strain 0154; *Grifola frondosa* (Dicks.: Fr.) S.F. Gray, strain 0917; *Grifola umbellata* (Pers.) Pilát, strain 1622; *Lentinula edodes* (Berk.) Pegler, strain F-249 from the collection of Mycology and Algology Department of Lomonosov Moscow State University (Moscow, Russia), *G. applanatum*, strain SIE1304; *Ganoderma colossus* (Fr.) C.F. Baker, strain SIE1301; *G. lucidum*, strain SIE1303; *Ganoderma neojaponicum* Imazeki, strains SIEbgm and SIEbidoup; *Tomophagus cattienensis* X.T. Le & Moncalvo, strain SIE1302 from the collection of Department of Botany, Southern Institute of Ecology (Ho Chi Minh City, Vietnam), *Ganoderma valesiacum* Boud., strain 120702; *Laetiporus sulphureus* (Bull.: Fr.) Murrill, strain 120707 from the collection of Botany Department of Irkutsk State University (Irkutsk, Russia), *L. edodes*, strain 198; *Pleurotus ostreatus* (Jacq.) P. Kumm., strain 69 from the collection of Experimental Mycology and Biodeterioration Laboratory, Institute of Microbiology, NAS of Belarus (Minsk, Republic of Belarus); *P*. *ostreatus*, strains BK1702 and HK352 (IBPPM RAS, Saratov, Russia). All stock cultures were maintained on wort (4 Brix) agar slants, subcultured several times a year, and stored at 4 °C. To obtain the fungal preparations, mushroom mycelia were grown in a submerged culture on synthetic media with glucose and yeast extracts for 21 days at 26 °C. To prepare the solid media, 2% (*m*/*v*) agar was added to the corresponding nutrient solutions. For fungal inoculum preparation, all fungal strains were initially grown on the wort agar medium in a Petri dish, then 0.5 cm-discs covered with mycelium from the agar plate culture were punched out with a self-designed cutter and transferred into the seed medium. The seeding mycelium for the submerged culture was grown on liquid media and inoculated at a level of 10% (*v*/*v*) into the freshly prepared autoclaved nutrient liquids. Then submerged growth was performed in a 250 mL flask containing 80 mL of the medium. Biopolymeric compositions were prepared on the basis of the extracellular metabolites of the basidiomycetes grown by means of the submerged cultivation technique on synthetic media for 30 days at 26 °C [100]. Briefly, a filtered, cell-free, EPS-containing liquid supernatant was sedimented by bringing the final concentration of added ethanol to 75% (*v*/*v*). The mixture was left to stand for 48 h, and the EPS sediment was separated by centrifugation (10,000× *g*), resuspended in 96% (*v*/*v*) ethanol, and dried in the air. AN analysis of minor chemical component levels in the EPS preparations was conducted using gas chromatographic analysis in the mass-spectrometric detection mode (GC-MS) using a Trace GC-DSQ mass spectrometer system (Thermo Finnigan, San Jose, CA, USA) [101]. Chromatographic analysis was also performed with a Shimadzu HPLC system (Shimadzu Corporation, Kyoto, Japan) in high-performance liquid chromatography (HPLC) mode to detect the biomolecules of mushroom cultures involved in the manufacture of biopreparations [102]. The effect of fungal EPSs was studied with respect to the growth and development of soft wheat plants (*Triticum aestivum* L.) of the Saratovskaya 29 variety.

### 4.2. Plant Material and Treatment

Healthy, visibly undamaged seeds of common spring wheat (*Triticum aestivum* L. cv. Saratovskaya 29; Federal Center of Agriculture Research of the South-East Region, Saratov) were treated with an aqueous solution of a commercial household detergent, washed with tap water, surface sterilized in 70% (*v*/*v*) ethanol for 60 s, treated with a two-fold diluted aqueous diacide (cetylpyridinium chloride, 660 mg/L; ethanol mercury chloride, 330 mg/L) for 4 min, and washed repeatedly with sterile distilled water. Seeds were germinated on distilled water-wetted sterile filter paper in Petri dishes for 1 day in the dark at 26 °C. Approximately 80 seeds per experimental treatment with each EPS solution were placed on glass rods in plastic cuvettes containing sterile distilled water and left in a thermostat at 25 °C for 2 days without lighting. Then, the etiolated 3-day-old seedlings were incubated in solutions of mushroom EPSs. For that, a distilled water medium in each plastic cuvette was replaced with an aqueous solution of the fungal preparation (each used at a final concentration of 15, 40, and 80 mg/L), and the seedlings continued to grow for another 7 days under controlled conditions (temperature of 24 °C; air humidity of 60%; and 16-h daily illumination with a light intensity of 60 μM × m^−2^ × s^−1^). Cultures without the EPS fortification were considered the reference system. On the 10th day of the experiment, fresh biomass weight analysis was conducted, and weight data were recorded from 4 lots of 5 plants. The morphological variables of the seedlings were measured, alongside the biochemical parameters of the leaves.

### 4.3. Malondialdehyde Measurement

The lipid peroxidation level was determined by the content of 2-thiobarbituric acid (TBA) reactive substances (TBARSs), with the overall content of TBARSs being expressed in terms of MDA in accordance with a modified technique [103,104]. MDA was determined spectrophotometrically by coloration developed in the course of MDA-TBA adduct formation at elevated temperatures. To this end, 100 mg of leaf tissue was homogenized in 1.5 mL of 20% (*m*/*v*) trichloroacetic acid, and the homogenate was centrifuged (10,000× *g*, 4 °C, 15 min). Then, 0.3 mL of the liquid supernatant was mixed with 1.2 mL of the 0.5% (*m*/*v*) TBA solution in 20% (*m*/*v*) trichloroacetic acid. The reaction mixture was heated in a water bath (95°, 30 min), cooled immediately, and recentrifuged (10,000× *g*, 4 °C, 5 min), and the absorbance of this mixture was recorded at λ = 532 nm and λ = 600 nm. The MDA concentration was calculated, taking into account a molar extinction coefficient (156 mM^−1^ × cm^−1^). Samples were examined in four analytical replicates, with three samples in each replicate.

### 4.4. Total Phenol Content Measurement

Total phenolics were extracted by ethanol (80%, *v*/*v*) [105]. The Folin–Ciocalteu method was employed to determine the total content of phenolics as the equivalent of gallic acid (GAE) used as a reference standard, by means of a combined technique [106,107]. Briefly, 50 μL of the sample and 50 μL of the Folin–Ciocalteau phenol reagent [108] were pipetted into an Eppendorf tube. The contents were vortexed for 10 s and then left to stand at room temperature for 2 min before the reaction was stopped by adding 500 μL of a 5% (*w*/*v*) sodium carbonate solution and 400 μL of distilled water, and the volume was adjusted to 1 mL. The mixture was then vortexed and incubated at 45 °C for 30 min before cooling rapidly. The absorbance of the solution was measured at λ = 760 nm. Gallic acid concentrations ranging from 25 to 300 mg/L were prepared, and a calibration curve was obtained using a linear fit. The amount of phenolics in the samples was expressed as GAE. Samples were examined in four analytical replicates, with three samples in each replicate.

### 4.5. Hydrogen Peroxide Measurement

Hydrogen peroxide was determined by a method implementing xylenol orange using a combined technique [109,110,111] with modifications. Leaves were homogenized at 4 °C in a mortar in 25 mM phosphate buffer (pH 6.2; biomass-to-buffer ratio 1:5, g/mL) and centrifuged (10,000× *g*, 4 °C, 15 min). The reaction mixture contained 1 mL of a 0.00761% (*m*/*v*) xylenol orange solution in 1.82% (*m*/*v*) aqueous sorbitol; 50 μL of a 0.233% (*m*/*v*) (FeSO_4_∙(NH_4_)_2_SO_4_∙6H_2_O) solution in 5.81% (*v*/*v*) aqueous H_2_SO_4_; and 1 mL of the H_2_O_2_-containing solution (extract to be analyzed). This mixture was incubated at room temperature for 40 min and centrifuged (10,000× *g*, room temperature, 5 min), and the absorbance was measured at ƛ = 560 nm. Hydrogen peroxide concentrations ranging from 0.015 to 1.500 mg/L were prepared, and a calibration curve was obtained using a linear fit. The amount of H_2_O_2_ in the photometric cuvette was determined from this curve followed by recalculation in terms of the analyzed biomass extracts. Samples were examined in four analytical replicates, with three samples in each replicate.

### 4.6. Enzymatic Activity Determinations

Enzymes were extracted from biomass according to the combined technique [112,113,114] with some modifications. The collected and frozen plant material was ground to a fine powder in a chilled mortar. The enzymes were extracted using 10 mL (per 1 g of fresh weight) of cold 50 mM phosphate buffer (pH 7.5) containing 1 mM PMSF (phenylmethylsulfonyl fluoride, Sigma-Aldrich, St. Louis, MO, USA). The homogenates were centrifuged (10,000× *g*, 4 °C, 15 min), and the supernatant liquids were stored in an ice bath between the steps of the analysis. The enzymatic activity values were determined spectrophotometrically. Peroxidase activity was measured by the oxidation rate of 4,4′-diaminodiphenyl (benzidine) with H_2_O_2_ at ƛ = 590 nm (*ε* = 34,000 M^−1^ × cm^−1^) [115]. Superoxide dismutase activity determination was evidenced by the fact that *para*-nitroblue tetrazolium (NBT) is capable of being photochemically reduced into a blue-colored substance formazan in the presence of riboflavin. By neutralizing cellular reductants as superoxide (dioxygen radical anion) to yield hydrogen peroxide, SOD inhibits the above-mentioned reduction process [116]. The photochemical blue formazan production from NBT was monitored by measuring absorbance at ƛ = 560 nm, and the SOD activity was estimated as the enzyme amount required to inhibit the NBT photoreduction [76]. In general, one unit of enzymatic activity was defined as the amount of enzyme required to catalyze the formation of 1 µmol of the catalytic reaction product or the disappearance of 1 µmol of the analyzed enzyme substrate per min, and the activity was expressed in terms of the protein content. Protein quantification was conducted using the technique developed by M.M. Bradford [117]. Samples were examined in four analytical replicates, with three samples in each replicate.

### 4.7. Statistical Analysis

Data were processed by a one-way dispersion analysis ANOVA. To compare the averages for the experimental treatments, the statistical significances of the difference between means were determined using Duncan’s test at a significance level of 95% (*p* ≤ 0.05) using the program package Statistica 10 (StatSoft Inc., St. Tulsa, OK, USA). The ranking according to Duncan’s test is indicated in the tables by different Latin letters. The figures depict the mean values with standard deviations. The obtained physiological and biochemical data were subjected to statistical analysis using a Fisher test to assess the significance differences, with the outcomes depicted in the figures by asterisks as follows: * *p* ≤ 0.05; ** *p* ≤ 0.005; *** *p* ≤ 0.0005; **** *p* < 0.0001. Data on the morphological, physiological, and biochemical variables of the plants were obtained in three independent experiments.

## 5. Conclusions

Although microbial-based plant biostimulants have been widely used in agriculture, many scientific issues remain unresolved, with one of the principal questions being related to any evidence of xylotrophic basidiomycetes’ participation. This is the first study to have found how the xylotrophic basidiomycetes’ exopolysaccharides function in the growth stimulation and antioxidant protection of wheat plants. This function consists of regulation of the activity of antioxidant enzymes (peroxidase and superoxidedismutase), leading to a decrease in the leaf content of MDA. POx activity was upregulated by six preparations (*p* ≤ 0.005), and the H_2_O_2_ concentration was maintained near the baseline in the majority of experimental modes, and even decreased in plants exposed to approximately half of the preparations used (*p* ≤ 0.05). The concerted impact on these variables is capable of reducing oxidative damage. As a result, the EPS-treated seedlings grew better compared to the untreated controls. In summary, 15 of the 29 preparations showed positive effects in distinct terms regarding the effect on one or even all morphological parameters studied. These cases are marked in yellow in Table 1 and Table S1 and are sourced from the basidiomycetes *F. velutipes*, *G. colossus*, *G. neojaponicum* SIEbgm, *G. neojaponicum* SIEbidoup, *Gr. umbellata*, *Laet. sulphureus*, *L. edodes* 198, *L. edodes* F-249, *P. ostreatus* 69, and *T. cattienensis*. It could therefore be hypothesized that basidiomycetes’ EPSs might serve as beneficial exogenous polymeric substances capable of inducing improved wheat plant growth and alleviating the environmental stress resistance mediated by initiating adequate antioxidant responses in wheat. However, such questions can be answered through extensive research on stress alleviation by fungal EPSs, which needs to be further investigated. Fungal exopolysaccharides constitute a significant yet unexplored category of natural compounds with potential positive impacts on plants. The results of this work encourage a more thorough exploitation of the potentialities of natural resources provided by basidiomycetous fungi and may present a prospect for innovative applications for improved crop cultivation technology.

## Figures and Tables

**Table 1:** Morphometric variables * in 10-day-old wheat seedlings exposed to fungal exopolysaccharides.

Assay Mode	Fungal Producent	Applied Concentration, mg/L	Coleoptile Length, cm	Leaf Length, cm	Root Length, cm	Root Number
Control	Water	0	1.19 b–e	4.77 b–g	2.23 ef	2.20 b–h
1	*Armillaria mellea* 0738	40	1.09 a–d	4.71 b–f	2.45 e–g	2.20 b–h
2	*Armillaria mellea* 1346	40	0.98 ab	4.17 b–e	1.90 de	2.30 d–h
3	*Flammulina velutipes* 0535	40	0.95 a	6.39 h–j	3.51 hi	2.29 c–h
4	*Flammulina velutipes* 0535	80	1.07 a–c	5.30 e–i	1.88 de	1.77 a–d
5	*Ganoderma applanatum* 0154	40	1.17 b–e	4.90 c–g	1.73 c–e	1.72 a–c
6	*Ganoderma applanatum* SIE1304	40	0.92 a	3.53 ab	0.99 ab	1.77 a–d
7	*Ganoderma colossus* SIE1301	40	1.10 a–d	5.61 f–i	3.41 hi	2.24 c–h
8	*Ganoderma colossus* SIE1301	80	1.64 jk	5.27 d–i	1.38 b–d	3.00 ij
9	*Ganoderma lucidum* 1315	40	0.93 a	3.93 a–d	0.97 ab	1.94 a–e
10	*Ganoderma lucidum* SIE1303	40	1.18 b–e	4.46 b–f	1.45 b–d	2.56 f–i
11	*Ganoderma neojaponicum* SIEbgm	40	1.29 d–g	6.36 h–j	4.28 j	1.48 a
12	*Ganoderma neojaponicum* SIEbidoup	15	1.19 bcde	5.66 fghi	1.81 cde	1.87 abcd
13	*Ganoderma neojaponicum* SIEbidoup	40	1.69 jk	7.63 j	3.98 ij	3.20 jk
14	*Ganoderma neojaponicum* SIEbidoup	80	1.61 i–k	6.46 ij	3.20 h	3.20 jk
15	*Ganoderma valesiacum* 120702	40	1.21 c–e	5.61 f–i	2.24 ef	1.58 a
16	*Grifola frondosa* 0917	40	0.91 a	4.56 b–f	2.26 ef	2.01 a–f
17	*Grifola umbellata* 1622	40	1.54 h–j	6.55 ij	2.91 f–h	3.20 jk
18	*Grifola umbellata* 1622	80	1.21 c–e	6.12 g–i	2.36 ef	1.82 a–d
19	*Laetiporus sulphureus* 120707	40	1.49 g–j	5.05 c–h	1.86 de	3.00 ij
20	*Laetiporus sulphureus* 120707	80	1.35 e–h	3.82 a–c	0.99 ab	2.73 h–j
21	*Lentinula edodes* 198	40	1.37 e–h	4.37 b–f	1.11 a–c	3.13 j
22	*Lentinula edodes* F-249	40	0.91 a	6.52 ij	3.16 h	2.16 b–g
23	*Lentinula edodes* F-249	80	1.16 b–e	5.34 e–i	3.06 gh	2.44 e–h
24	*Pleurotus ostreatus* 69	15	1.04 a–c	4.58 b–f	1.33 b–d	1.67 ab
25	*Pleurotus ostreatus* 69	40	1.79 k	6.57 ij	3.12 gh	3.27 jk
26	*Pleurotus ostreatus* 69	80	1.43 f–i	5.55 f–i	3.38 hi	3.00 ij
27	*Pleurotus ostreatus* BK1702	40	0.90 a	4.11 b–e	0.53 a	2.59 g–i
28	*Pleurotus ostreatus* HK352	40	1.03 a–c	4.81 b–g	1.20 a–d	1.87 a–d
29	*Tomophagus cattienensis* SIE1302	40	1.25 c–f	2.80 a	0.95 ab	3.67 k

* Data were processed using one-way ANOVA. Latin letters indicate differences between treatments according to the results of Duncan’s test at *p* ≤ 0.05. n = 20.

**Table 2:** Pearson correlation coefficients (R) for morphological indices associated with biochemical variables of wheat plants exposed to selected fungal preparations.

Fungal Producent	Applied Concent- Ration, mg/L	Sample Group	Biochemical Variable	Coleoptile Length R (*p*-Value)	Leaf Length R (*p*-value)	Root Length R (*p*-Value)	Root Number R (*p*-Value)
Water	0	I	MDA level	ns *	ns	−0.2635 (0.2310)	ns
*Flammulina velutipes* 0535	40						
*Ganoderma colossus* SIE1301	40		Phenol content	−0.2619 (0.2324)	−0.5485 (0.0503)	−0.7406 (0.0071)	+0.4087 (0.1205)
*Ganoderma neojaponicum* SIEbgm	40						
*Ganoderma neojaponicum* SIEbidoup	40		H_2_O_2_ concent- ration	−0.4129 (0.1178)	+0.3527 (0.1588)	+0.2330 (0.2586)	−0.4308 (0.1069)
*Grifola umbellata* 1622	40						
*Laetiporus sulphureus* 120707	40		Pox activity	ns	+0.4567 (0.0923)	+0.5058 (0.0679)	+0.2818 (0.2151)
*Lentinula edodes* F-249	40						
*Pleurotus ostreatus* 69	40		SOD activity	+0.3671 (0.1483)	ns	ns	+0.4520 (0.0948)
*Tomophagus cattienensis* SIE1302	40						
Water	0	II	MDA level	−0.5403 (0.0834)	ns	−0.3540 (0.1948)	−0.7244 (0.0211)
*Flammulina velutipes* 0535	80						
*Ganoderma colossus* SIE1301	80		Phenol content	ns	−0.6455 (0.0419)	−0.8190 (0.0064)	ns
*Ganoderma neojaponicum* SIEbidoup	80						
			H_2_O_2_ concent- ration	ns	+0.4840 (0.1121)	+0.3505 (0.1973)	ns
*Grifola umbellata* 1622	80						
*Laetiporus sulphureus* 120707	80		Pox activity	+0.4173 (0.1518)	+0.5751 (0.0679)	+0.7543 (0.0153)	+0.4810 (0.1138)
*Lentinula edodes* F-249	80		SOD activity	+0.4592 (0.1262)	+0.4099 (0.1566)	+0.7135 (0.0234)	+0.4194 (0.1505)
*Pleurotus ostreatus* 69	80						
Water	0	III	MDA level	−0.9406 (0.1103)	−0.9454 (0.1056)	−0.9712 (0.0766)	−0.9933 (0.0368)
*Ganoderma neojaponicum* SIEbidoup	15		Phenol content	−0.6287 (0.2836)	ns	ns	−0.4343 (0.3570)
			H_2_O_2_ concent- ration	+0.8660 (0.1667)	+0.9881 (0.0492)	+0.9980 (0.0403)	+0.9574 (0.0933)
*Pleurotus ostreatus* 69	15		Pox activity	+0.6287 (0.2836)	ns	ns	+0.4343 (0.3570)
			SOD activity	+0.9962 (0.0277)	+0.8303 (0.1882)	+0.8776 (0.1591)	+0.9897 (0.0457)

* ns is for R < 0.2, not significant at a probability level of 0.05.

## Data Availability

All data are available within the manuscript.

## Supplementary Materials

The following supporting information can be downloaded at: https://www.mdpi.com/article/10.3390/ijms25136877/s1.
