# Supplementary material for: Basidiomycetes Polysaccharides Regulate Growth and Antioxidant Defense System in Wheat
Source: Int J Mol Sci. 2024 Jun 22;25(13):6877. doi: 10.3390/ijms25136877 (PMC11241571; doi:10.3390/ijms25136877)
Supplement: Supplementary file 1 [file ijms-25-06877-s001.zip › ijms-3036902-supplementary.pdf]

Table S1. Relative values of the morphometric variables* in 10-day-old wheat seedlings exposed to fungal exopolysaccharides.

Assay mode	Fungal producent	Applied concentration, mg/L	Coleoptile length, % of control	Leaf length, % of control	Root length, % of control	Root number, % of control
Control	Water	0	100.00 b-e	100.00 b-g	100.00 gh	100.00 d-h
1	*Armillaria mellea* 0738	40	92.01 a-d	98.74 b-g	110.08 hi	100.00 d-h
2	*Armillaria mellea* 1346	40	82.64 ab	87.37 a-e	85.34 d-h	104.55 f-h
3	*Flammulina velutipes* 0535	40	79.70 a	133.82 h-j	157.36 jk	103.92 f-h
4	*Flammulina velutipes* 0535	80	89.79 a-c	110.99 c-i	84.50 d-h	80.43 a-d
5	*Ganoderma applanatum* 0154	40	98.24 b-e	102.75 b-h	77.45 b-h	78.26 a-c
6	*Ganoderma applanatum* SIE1304	40	77.78 a	74.03 ab	44.44 ab	80.43 a-d
7	*Ganoderma colossus* SIE1301	40	92.98 a-d	117.53 d-i	153.20 jk	101.96 e-h
8	*Ganoderma colossus* SIE1301	80	138.20 jk	110.34 c-i	71.36 b-g	136.36 lm
9	*Ganoderma lucidum* 1315	40	78.37 a	82.23 abc	43.34 ab	88.24 a-f
10	*Ganoderma lucidum* SIE1303	40	99.31 b-e	93.39 b-f	65.00 b-f	116.36 h-j
11	*Ganoderma neojaponicum* SIEbgm	40	108.71 d-g	133.19 h-j	192.08 lm	67.39 a
12	*Ganoderma neojaponicum* SIEbidoup	15	100.30 b-e	118.64 e-i	81.28 c-h	84.78 a-f
13	*Ganoderma neojaponicum* SIEbidoup	40	142.13 jk	159.92 i	205.87 m	145.45 m
14	*Ganoderma neojaponicum* SIEbidoup	80	135.39 i-k	135.34 ij	165.49 j-l	145.45 m
15	*Ganoderma valesiacum* 120702	40	102.10 c-f	117.59 d-i	90.59 e-h	71.74 ab
16	*Grifola frondosa* 0917	40	76.88 a	95.50 b-f	101.57 gh	91.30 b-g
17	*Grifola umbellata* 1622	40	129.78 h-j	137.29 ij	150.64 jk	145.45 lm
18	*Grifola umbellata* 1622	80	101.80 c-f	128.17 g-i	106.04 gh	82.61 a-e
19	*Laetiporus sulphureus* 120707	40	125.84 g-j	105.87 b-i	95.97 f-h	136.36 k-m
20	*Laetiporus sulphureus* 120707	80	113.48 e-h	80.03 a-c	50.91 a-d	124.24 i-l
21	*Lentinula edodes* 198	40	115.17 e-h	91.48 b-e	57.17 a-e	142.42 lm
22	*Lentinula edodes* F-249	40	77.04 a	136.62 ij	141.95 jk	98.04 c-h
23	*Lentinula edodes* F-249	80	97.91 b-e	111.82 c-i	137.42 ij	110.91 g-i
24	*Pleurotus ostreatus* 69	15	87.69 a-c	96.02 b-f	59.81 b-e	76.09 ab
25	*Pleurotus ostreatus* 69	40	150.56 k	137.71 ij	161.47j-l	141.70 lm
26	*Pleurotus ostreatus* 69	80	119.42 f-i	124.17 f-i	174.46 kl	136.36 j-m
27	*Pleurotus ostreatus* BK1702	40	75.90 a	86.19 a-d	23.86 a	117.65 h-k
28	*Pleurotus ostreatus* HK352	40	87.09 a-c	100.73 b-g	53.89 a-d	84.78 a-f
29	*Tomophagus cattienensis* SIE1302	40	105.62 c-f	58.66 a	49.25 a-c	166.67 n

* Data were processed by one-way ANOVA. Latin letters indicate differences between treatments according to the results of the Duncan's test at $p \leq 0.05$. n = 20.

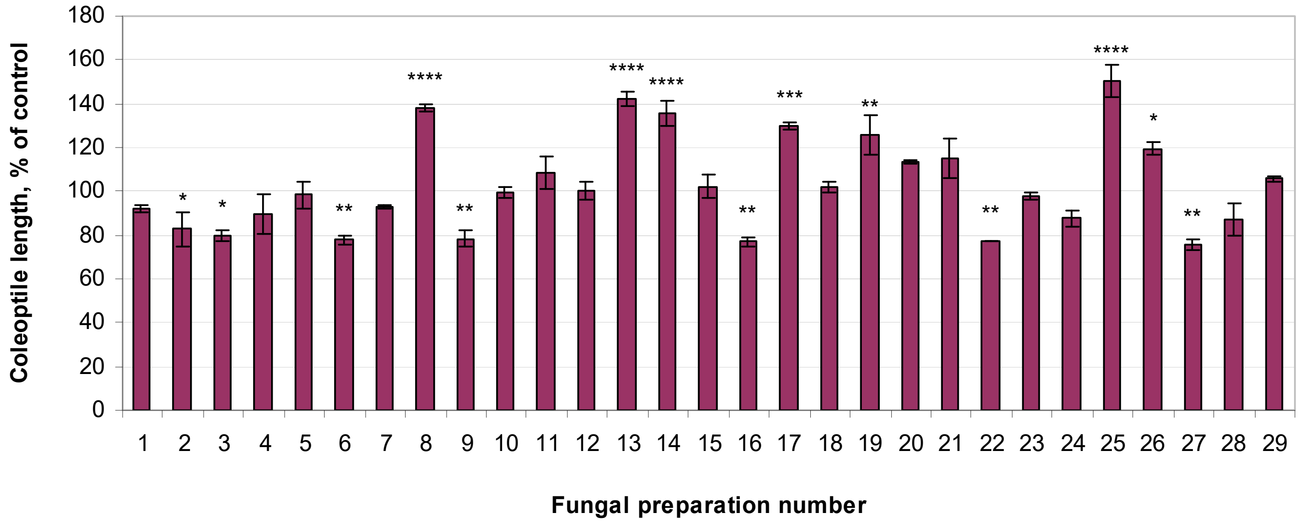

Figure S1. Relative coleoptile length values in 10-day-old wheat seedlings exposed to fungal preparations. EPS numbers correspond to those in Table 1. Values are means ± SD; *p≤ 0,05; **p≤ 0,005; ***p≤ 0,0005; ****p< 0,0001.

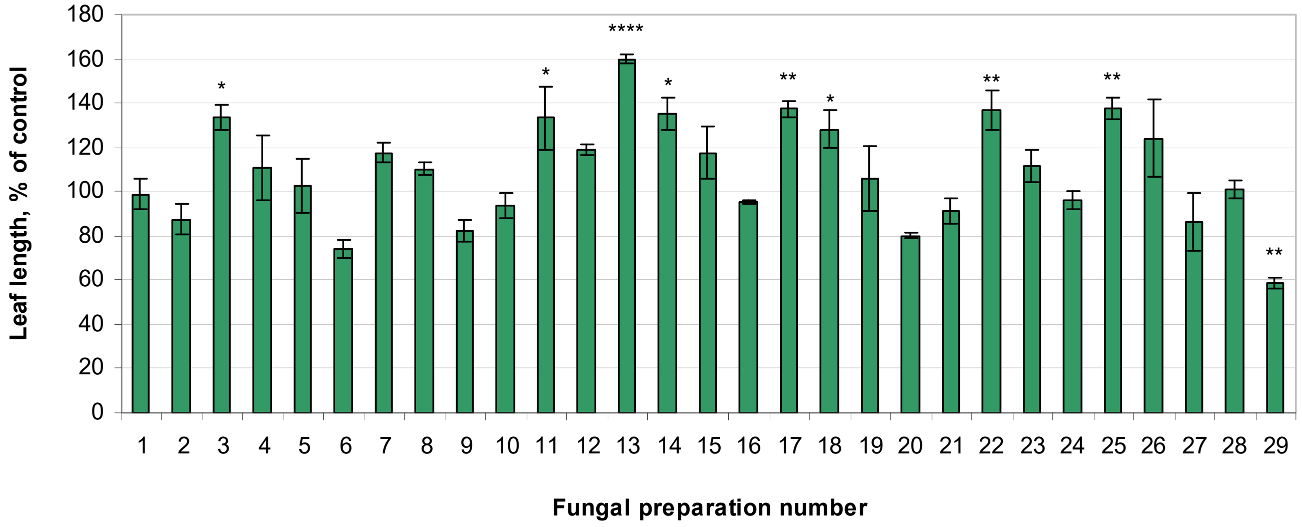

Figure S2. Relative leaf length values in 10-day-old wheat seedlings exposed to fungal preparations. EPS numbers correspond to those in Table 1. Values are means ± SD; *p≤ 0,05; **p≤ 0,005; ***p≤ 0,0005; ****p< 0,0001.

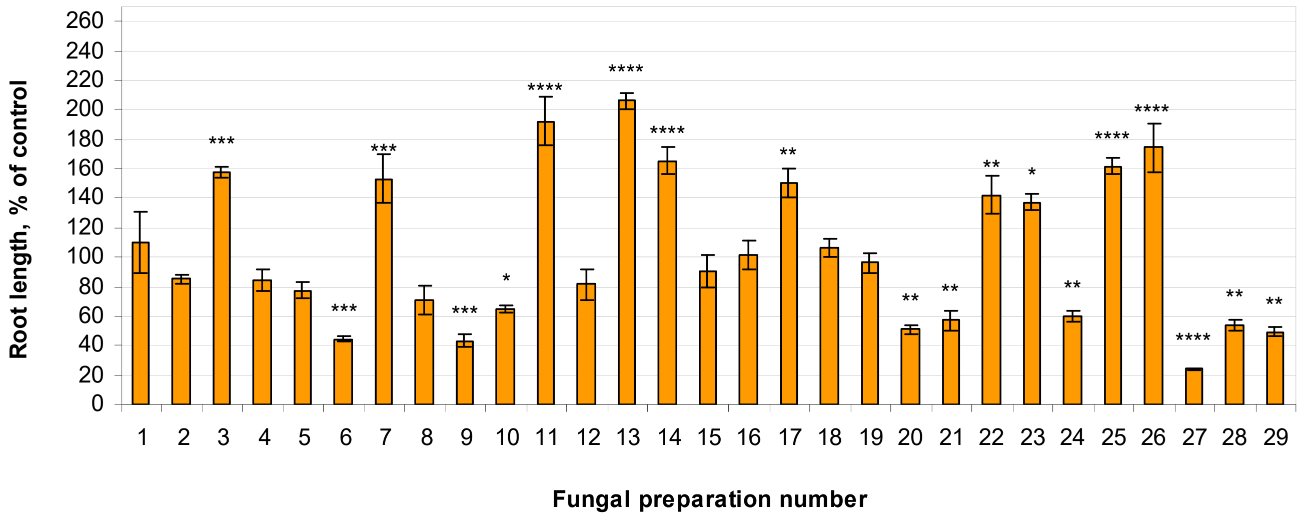

Figure S3. Relative root length values in 10-day-old wheat seedlings exposed to fungal preparations. EPS numbers correspond to those in Table 1. Values are means ± SD; *p≤ 0,05; **p≤ 0,005; ***p≤ 0,0005; ****p< 0,0001.

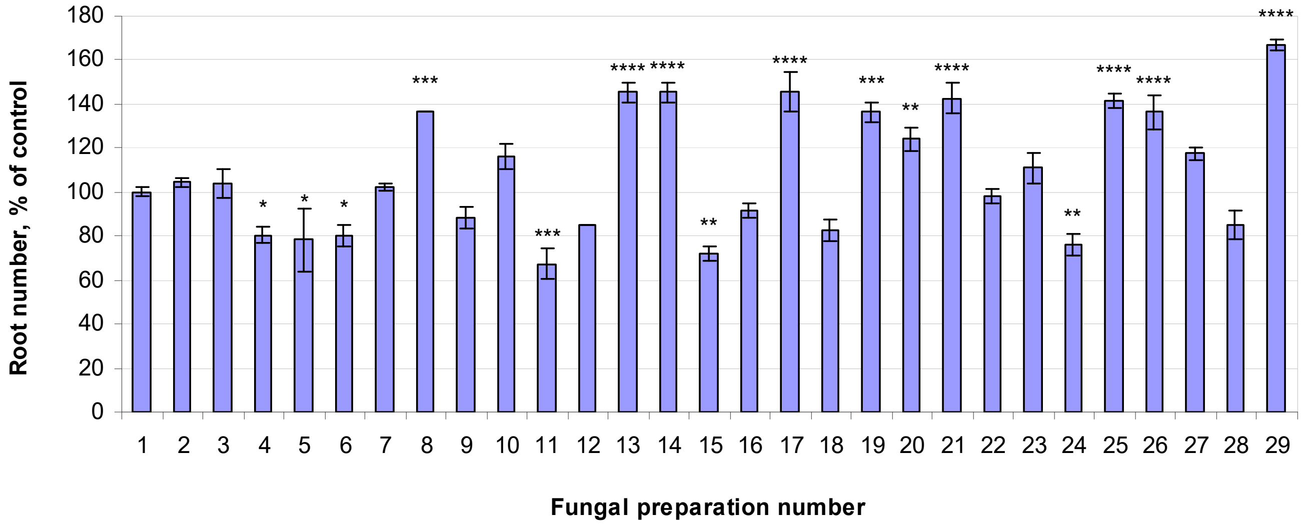

Figure S4. Relative root number values in 10-day-old wheat seedlings exposed to fungal preparations. EPS numbers correspond to those in Table 1. Values are means ± SD; *p≤ 0,05; **p≤ 0,005; ***p≤ 0,0005; ****p< 0,0001.
